# Unveiling the antibacterial mechanism of resveratrol against *Aeromonas hydrophila* through proteomics analysis

**DOI:** 10.3389/fcimb.2024.1378094

**Published:** 2024-03-06

**Authors:** Yuying Fu, Lishan Zhang, Yi Lin, Xinrui Zhao, Haoyu Chen, Yicheng Zhong, Wenjia Jiang, Xiaoyun Wu, Xiangmin Lin

**Affiliations:** ^1^ School of Safety and Environment, Fujian Chuanzheng Communications College, Fuzhou, China; ^2^ Fujian Provincial Key Laboratory of Agroecological Processing and Safety Monitoring (School of Life Sciences, Fujian Agriculture and Forestry University), Fuzhou, China; ^3^ Key Laboratory of Crop Ecology and Molecular Physiology (Fujian Agriculture and Forestry University), Fujian Province University, Fuzhou, China; ^4^ Ningde Customs, Fujian, Ningde, China; ^5^ Key Laboratory of Marine Biotechnology of Fujian Province, Institute of Oceanology, Fujian Agriculture and Forestry University, Fuzhou, China

**Keywords:** antibiofilm, resveratrol, *Aeromonas hydrophila*, quantitative proteomics, scan electron microscopy

## Abstract

This investigation delves into elucidating the mechanism by which resveratrol (Res), a natural polyterpenoid renowned for its antimicrobial properties, exerts its effects on *Aeromonas hydrophila*, a ubiquitous waterborne pathogen. Our findings underscore the dose-dependent manifestation of resveratrol in exhibiting antibacterial and antibiofilm formation activities against *A. hydrophila*. Employing a Data-independent acquisition (DIA) based quantitative proteomics methodology, we systematically compared differentially expressed proteins in *A. hydrophila* subjected to varying concentrations of Res. Subsequent bioinformatics analyses revealed key proteins and pathways pivotal in resveratrol’s antimicrobial action, encompassing oxidative stress, energy metabolism, and cell membrane integrity. Validation of the proteomics outcomes was meticulously conducted using the qPCR method at the mRNA level. Dynamic trend analysis unveiled alterations in biological processes, notably the correlation between the cell division-related protein ZapC and resveratrol content. Furthermore, scanning electron microscopy corroborated a significant elongation of *A. hydrophila* cells, affirming resveratrol’s capability to inhibit cell division. In concert, resveratrol emerges as a participant in the cell membrane integrity pathway, biofilm formation, and potentially, the regulation of genes associated with cell division, resulting in morphological elongation. These revelations position resveratrol as a promising natural alternative to conventional antibiotics for treating *A. hydrophila* infections.

## Introduction

*Aeromonas hydrophila (A.h)*, a Gram-negative bacterium prevalent in aquatic environments, poses zoonotic risks and exhibits varying pathogenicity in aquatic animals and mammals (Martins et al., 2002). Antibiotic treatment has been a primary strategy to combat *A. hydrophila* infections in aquaculture; however, the escalating prevalence of drug-resistant strains due to antibiotic misuse necessitates urgent attention (Peng et al., 2017, Peng et al., 2021). For instance, strains isolated by Tartor et al. from Nile tilapia displayed resistance to multiple antibiotics, emphasizing the urgency of addressing this issue (Tartor et al., 2021). As a result, fish farmers have no choice but to increase antibiotic doses to treat diseases in aquaculture, which poses a serious threat to public health and environmental safety. In addition, disease control through the administration of antibiotics without adequate evaluation of the effectiveness of treatment also leads to the infiltration of residues into the environment and the destruction of the microbiome in the area (Liu et al., 2020).

In this context, the exploration of alternative antimicrobial agents becomes imperative.

Resveratrol (Res), a non-flavonoid polyphenol organic compound found in various plants, presents itself as a potential solution. With documented antioxidant, anti-inflammatory, and antibacterial properties, resveratrol exhibits diverse therapeutic effects. For example, resveratrol can reduce intestinal damage and dysfunction in heat-stressed rats by down-regulating the expression and activity of oxidase and inhibiting NADPH oxidase-mediated ROS production to improve oxidation status and inhibit inflammatory response (Cheng et al., 2019). Notably, its antibacterial activity against foodborne pathogens has been established, including strains such as *Staphylococcus aureus* (Lai et al., 2017), *Bacillus cereus* (Promgool et al., 2014), *Bacillus subtilis* (Kumar et al., 2012) and *Listeria monocytogenes* (Ferreira and Domingues, 2016) and *E. coli* (Seukep et al., 2016), etc. Despite these attributes, the specific antibacterial mechanisms of resveratrol against *A. hydrophila* remain elusive. To address this knowledge gap, we employed a quantitative proteomics approach, combining data-independent acquisition (DIA) with bioinformatics analysis. This comprehensive methodology enables a systematic examination of protein expression changes, offering insights into the potential antibacterial mechanisms of resveratrol against *A. hydrophila*.

## Materials and methods

### Bacterial strain and regents

*A. hydrophila* ATCC 7966 (*A.h*) was kindly provided by Prof. Peng in Sun Yat-Sen university, its suitable incubated in Luria-Bertani culture medium at 30°C. Res (CAS No. 501-36-0, 99.0% purity) was purchased from Rhawn company, stock solution was prepared by the volume ratio of 3% DMSO.

### Effect of Res on the growth of *A. hydrophila*

The effect of resveratrol on the growth of *A. hydrophila* was assessed using a slightly modified version of the previously established method (Zhang et al., 2020). Briefly, *A. hydrophila* was transferred to 300 µL Luria-Bertani (LB) medium with final concentrations of 0, 16, 32, 64, and 128 µg/mL Res in a ratio of 1:100. Subsequently, the optical density (OD600) at 600 nm was measured using an automatic growth curve analyzer (Bioscreen C, Helsinki, Finland).

### Protein extraction and trypsin digestion

After an overnight incubation, *A. hydrophila* strains were transferred to 30 mL LB medium at a ratio of 1:100 with 48 or 64 μg/mL Res, respectively, and cultured in a 30°C shaker till OD600 = 1.0. The cells were harvested by centrifugation at 6000g for 10 min at 4°C. The cell pellets were washed three times with PBS buffer and lysed by sonication in lysis buffer (6 M urea, 2 M thiourea, 100 mM Tris–HCl pH 8.5, protease inhibitor). The samples were disrupted on ice for 15 min and then centrifuged at 12000x g for 20 min Subsequently, the supernatant containing the whole protein was collected, and its concentration was determined using the Bradford method. 50μg of the protein sample was obtained for enzymatic hydrolysis using filter-aided sample preparation (FASP) method (Wiśniewski, 2017). The protein was subjected to reduction and alkylation using dithiothreitol (DTT) and iodoacetamide (IAA), as previously described (Zhang et al., 2023). The protein was digested with trypsin (the volume ratio of trypsin to sample was 1:50) for 16 h at 37°C. After digesting, the peptides were desalted and dried using Sep-Pak Vac C18 Column (Waters Inc., Milford, MA) and CentriVap Concentrator (Labconco, Inc., Kansas City, MO), respectively.

### Quantitative proteomics and data analysis

The digested peptides were resuspended in 0.1% formic acid (FA) and 2% acetonitrile (ACN) solution, and then the samples were separated using the EASY-nano-LC chromatographic system (Thermo Scientific Inc., Waltham, MA, USA) with identical parameters as previously described (Song et al., 2023). In brief, peptides were separated using solvent gradient chromatography with binary-mobile phase system of buffer A (2% ACN and 0.1% FA) and buffer B (98% ACN and 0.1% FA) as follows: 0-18 min, 6-12% B; 18-77 min, 12-20% B; 77-109 min, 20-32% B; 109-110 min, 32-90% B; 111-120 min, 90% B hold. The separated samples were subsequently analyzed using an Exactive HF mass spectrometer (Thermo Scientific Inc., Waltham, MA, USA) with an electrospray voltage of 2.0 kV and an ion source temperature of 320°C. The raw data obtained from data dependent acquisition (DDA) was imported into Spectronout Pulsar X to construct a DDA spectrum library. Subsequently, the DIA raw data obtained were imported into Spectronout Pulsar X and matched against the *A. hydrophila* ATCC 7966 database for qualitative and quantitative analysis of proteins. In the quantitative process, iTR calibration involved nonlinear fitting. A precursor Q-value cut off of 0.01 and a protein Q-value cut off of 0.01 were employed for protein identification purposes. For protein quantification, the peak area of daughter ions was utilized, with at least three daughter ions being selected to determine their average intensity.

### Bioinformatics analysis

The biological processes and major central metabolic pathways of differentially expressed proteins (DEPs) were classified and annotated of their Gene ontology (GO) annotations, and Kyoto Encyclopedia of Genes and Genomes (KEGG) pathways were analysis by DAVID (https://david.ncifcrf.gov/) (Wang et al., 2023a). To further investigate patterned differences in expression profiles with resveratrol, the dynamic trend analysis of DEPs was performed by clustering trend tools in Hiplot (https://hiplot.com.cn/).

### qPCR assay

The bacterial strains were transferred to LB medium containing 3% DMSO and either 48 or 64 μg/mL Res, respectively. The cultures were grown until reaching an optical density at 600 nm (OD600nm) of 1.0, and RNA was immediately extracted using TRIzol (Thermo Fischer Scientific, Walthan, MA, USA) (Jiang et al., 2022). Briefly, the bacterial liquid of each group was centrifuged at 9000 rpm for 10 min, and then homogenized with 1 mL TRIzol. Subsequently, 200 μL chloroform was added to the mixture, centrifuged at 11,000 rpm for 10 min. The clear upper layer containing RNA was carefully transferred to a new 1.5 mL tube, where it was mixed with 500 μL isopropyl alcohol. After incubating at room temperature for 20 min, the mixture was centrifugation at 10000 rpm for 10 min. Total RNA isolated was washed twice with 75% ethanol before being eluted in 200 μL nuclease-free water. Finally, the isolated RNA were transferred to cDNA using the Takara reverse transcription kit, followed by qPCR amplification according to the manufacturer’s instructions (Zhou et al., 2024). The primer sequences used in this study are provided in **Table S1**.

### Scanning electron microscopy (SEM) analysis

The morphology in *A. hydrophila* with or without Res treatment was investigated by conducting SEM using previously described methods (Guo et al., 2018). Briefly, the overnight cultured strain culture was transferred to 5 mL LB medium containing 3% DMSO, 48 or 64 μg/mL Res, respectively, and then cultured 3 h at 30°C shaker. The strains were centrifuged at 2000 g for 10 min and the pellet was fixed in 2.5% glutaraldehyde (pH 7.4) for 2h. After being washed three times with phosphate buffer (0.1 M, pH 7.2), the samples were fixed in 1% osmic acid solution at 4°C for 2 h. Following this step, gradient dehydration using ethanol was performed until critical point drying could be achieved. Finally, gold sputter-coating was applied to the samples for approximately thirty seconds before observation under a HITACHI Regulus8100 scanning electron microscope.

### Biofilm formation assay

Crystal violet staining was used to evaluate resveratrol’s antibiofilm activity, as previously described with slightly modification (Li et al., 2023). Briefly, overnight cultured bacterial strain was transferred to 96 microwell plates containing series of concentrations of Res in LB medium at a volume ratio of 1:20. Following incubation at 30°C for 24 h, the plates were washed twice with water until all floating cells were completely removed. Subsequently, 5% crystal violet dyeing solution was added and incubated for 20 min before being discarded and washed twice prior to drying. The absorbance at a wavelength of 595 nm was measured using SpectraMax i3 (Molecular Devices Co., Ltd., Shanghai, China) after the addition of a 95% ethanol solution. The experiment was performed in triplicate independently.

## Results

### The antibacterial activity of resveratrol against *A. hydrophila*

**Figure 1:**
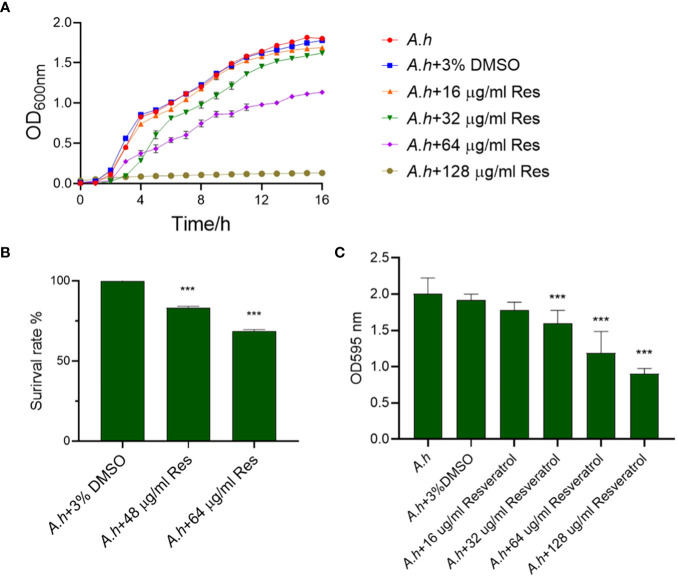
The effects of resveratrol on *A*. *hydrophila*. **(A)** The growth curves of *A*. *hydrophila* under serials of resveratrol concentrations treatments, respectively; **(B)** The survival capabilities of *A*. *hydrophila* in 48, 64 μg/mL resveratrol culture after 12 h incubation; **(C)** Effects resveratrol on the antibiofilm activity of *A*. *hydrophila*. The without Res and 3% DMSO treatment groups were performed both as negative controls in this study. *** represents P<0.05.

To assess the antibacterial activity of resveratrol against *A. hydrophila*, the growth curves of *A. hydrophila* treated in serials of concentrations of resveratrol were measured in this study. As showed in **Figure 1A**, the growth tendencies of bacterial strain in normal LB medium or 3% DMSO both as negative controls were almost the same. However, the growth curves were decreasing with the increase of the concentration of resveratrol. When compared to negative control that without treatment, the survival rates of *A. hydrophila* treated in 48 and 64 μg/mL resveratrol at 12 h were 82% and 68%, respectively, while almost 0% in 128 μg/mL resveratrol, which indicate Res can inhibit the growth of *A. hydrophila* significantly (**Figure 1B**). The antibiofilm activity of resveratrol against *A. hydrophila* was evaluated by crystal violet staining. As shown in **Figure 1C**, the results showed that Res can significantly inhibit the biofilm formation of *A. hydrophila* in a dose-dependent manner. With the increase of Res concentration, the effects were significant. When comparing with negative control group that without treatment, the biofilm formation had a significant decreased from 32, 64 to 128 μg/mL Res content, and the antibiofilm rate was 83.44, 62.23, and 47.14%, respectively.

### Quantitative proteomics of *A. hydrophila* between with and without resveratrol treatment

**Figure 2:**
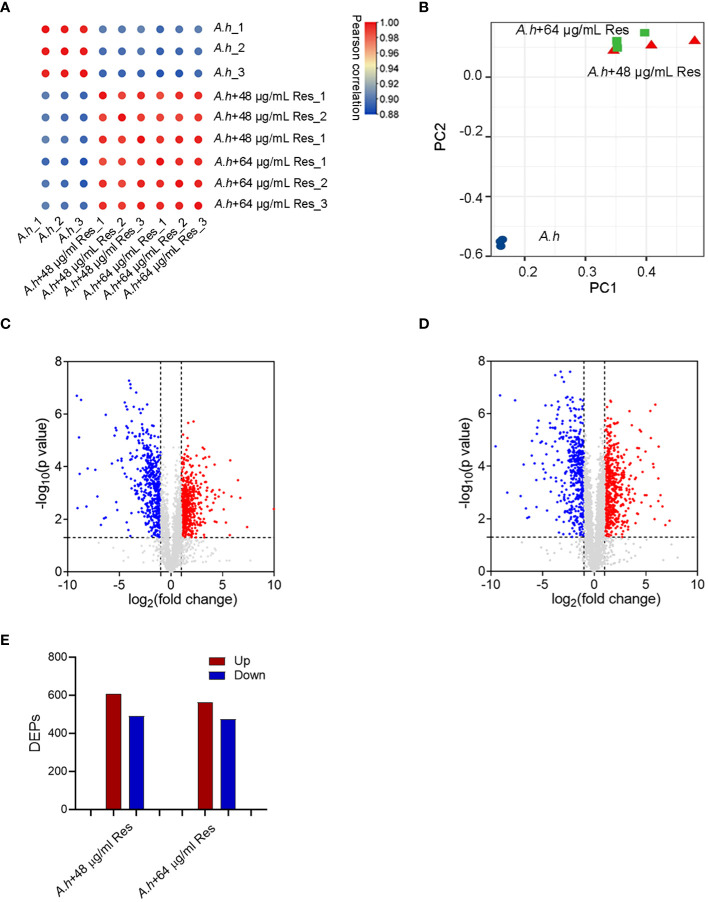
Quantitative proteomics analysis of DEPs among *A. hydrophilaI* with or without serials dose of resveratrol treatments. **(A)** Correlation analysis of protein abundance; **(B)** Principal component analysis (PCA). Different colors represent different samples and three dots of the same color represent three repeats; **(C, D)** The volcano plots of the significantly DEPs treated with 48, 64 μg/mL resveratrol, respectively. Each dot represents a protein, blue dot represents down regulated expression, red dot represents up regulated expression; **(E)** Histogram of numbers of DEPs in each comparison groups. The blue and red histogram represents down and up regulated expression, respectively.

To investigate the effect of Res, the DEPs among with or without 48 or 64 μg/mL Res treatment in *A. hydrophila* were compared by using quantitative proteomics method in this study. Three independent biological replicates were performed for each sample, ensuring the reproducibility and reliability of the experimental results, and the reliability of the mass spectrometry (MS) data was confirmed by calculating correlation coefficients among all samples, which higher than 0.88 (**Figure 2A**). The high correlation indicated the robustness and consistency of the data obtained. Moreover, PCA (principal component analysis) showed that two Res treated groups clustered closely and showed significant differences when compared with control (**Figure 2B**). By using LC-MS/MS analysis, a total of 3064 proteins were successfully identified in this study with a conservative threshold (protein and peptide false discovery rate <1%). Among these proteins, a total of 1098 DEPs were identified in the 48 μg/mL resveratrol stress condition, while 1036 DEPs were identified in the 64 μg/mL resveratrol stress condition (**Figures 2C, D**). Within the identified DEPs, 607 and 562 proteins displayed increasing expression in 48 μg/mL and 64 μg/mL resveratrol stress conditions, respectively, while 491 and 474 proteins exhibited decreasing expression in 48 μg/mL and 64 μg/mL resveratrol stress conditions, respectively (**Figure 2E**).

### Bioinformatics analysis

**Figure 3:**
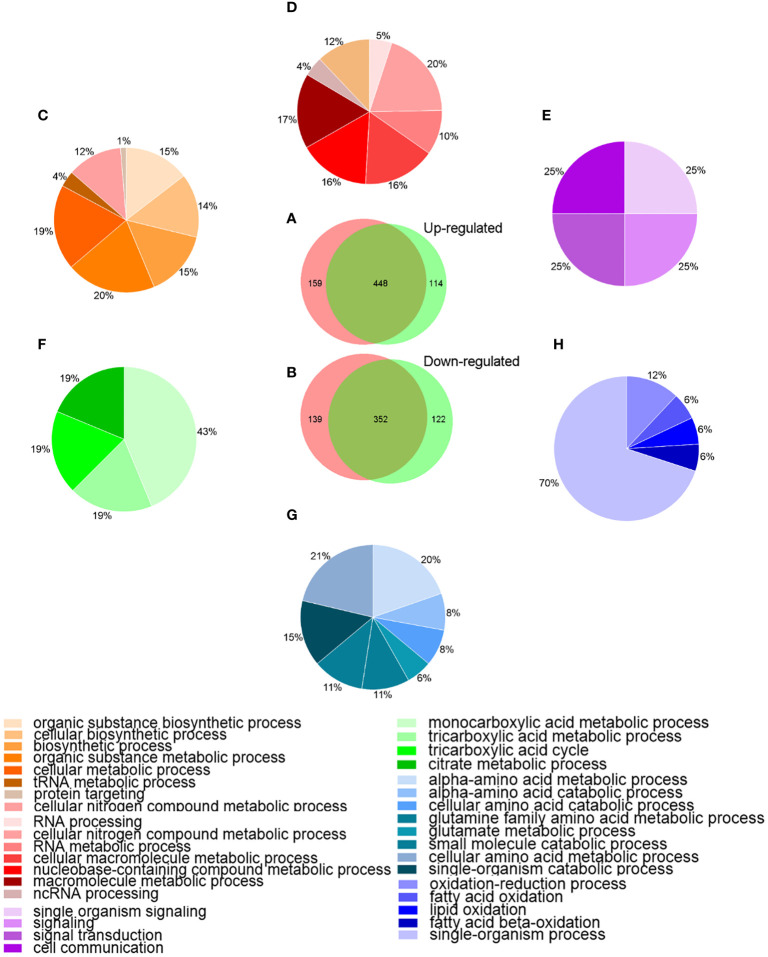
Venn-diagram and GO analysis of DEPs in *A. hydrophila* under 48, 64 μg/mL resveratrol treatment. **(A, B)** Venn-diagram showing the overlapping numbers of up and down-regulated proteins between *A.h*+48 μg/mL Res vs. *A.h*, and *A.*h+64 μg/mL Res vs. *A.h* group, respectively; **(C–E)** Biological processes related to the functional enrichment of up-regulated proteins among unique in 48 μg/mL Res group, overlap in both groups, and unique in 64 μg/mL Res group, respectively; **(F–H)** Biological processes related functional enrichment of down-regulated proteins among unique in 48 μg/mL Res group, overlap in both group, and unique in 48 μg/mL Res group, respectively. Different colors stand for different biological processes related to functional enrichments and were presented in those percentage pie charts accordingly.

To better understanding the behavior of *A. hydrophila* under Res stress, the DEPs among different groups were analyzed by bioinformatics method. Venn diagram and GO enrichment analysis were performed for common and unique altered proteins in each comparison groups. As showed in **Figure 3**, a total of 448 proteins were common up-regulated proteins between *A.h*+48 μg/mL Res vs. *A.h*, and *A.h*+64 μg/mL Res vs. *A.h* group, which involved in cellular nitrogen compound metabolic process, RNA metabolic process and cellular macromolecule metabolic process and other 8 biological processes. There were 159 unique up-regulated proteins in the *A.h*+48 μg/mL Res treatment group, which were involved in 11 biological processes, among which the top three GO terms were organic substance biosynthetic process, cellular biosynthetic process and biosynthetic process. 114 unique upregulated DEPs under 64 μg/mL Res treatment group were involved in 4 biological processes including single organism signaling, signaling, signal transduction and cell communication. There was a total of 352 common down-regulated proteins in *A.h*+48 μg/mL Res vs. *A.h* and *A.h*+64 μg/mL Res vs. *A.h* groups. These proteins are involved in 8 biological processes, among which the top 3 GO terms were α-amino acid metabolism, α-amino acid catabolism, and glutamine family amino acid metabolism. There were 139 unique down-regulated proteins in *A.h*+48 μg/mL Res group, which involved in monocarboxylic acid metabolism, tricarboxylic acid metabolism, tricarboxylic acid cycle and citric acid metabolism. In addition, the *A.h*+64 μg/mL Res group had 122 unique down-regulated proteins involved in 5 biological processes: oxidation-reduction process, fatty acid oxidation, lipid oxidation, fatty acid beta-oxidation and single-organism process.

**Figure 4:**
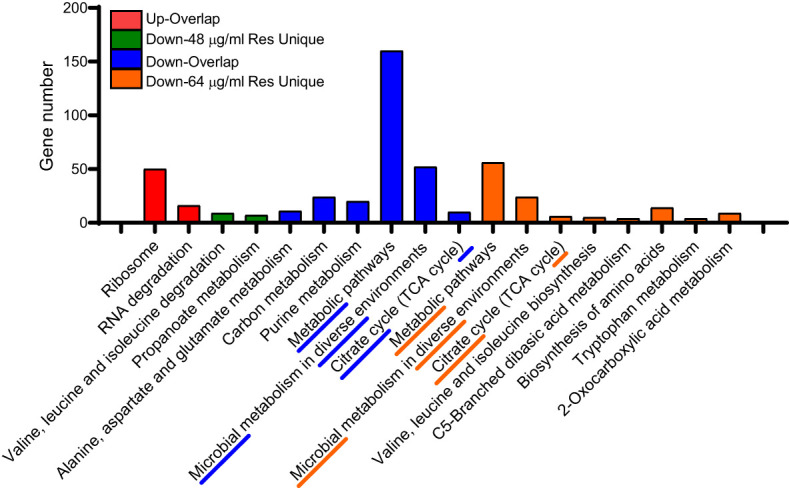
KEGG enrichment analysis of DEPs in *A. hydrophila* under 48, 64 μg/mL resveratrol, respectively. The top three enriched KEGG pathways of each comparison group are marked with underline and dashed lines.

We also performed KEGG enrichment analysis for the common and unique altered proteins between two comparison groups. As shown in **Figure 4**, the uniquedifferential expression of upregulated proteins in both comparisons failed to enrich significant metabolic pathways, and the common altered proteins were mainly involved in ribosome and RNA degradation. In addition, most of the common down-regulated DEPs are involved in carbon metabolism, purine metabolism, metabolic pathways and microbial metabolism in diverse fields environments and citrate cycle (TCA cycle). However, under 48 μg/mL resveratrol treatment, the unique down-regulated proteins are mainly involved in valine, leucine and isoleucine degradation and propanoate metabolism. Under the treatment of 64 μg/mL resveratrol, unique differentially expressed down-regulated proteins were mainly involved in metabolic pathways, microbial metabolism in diverse environments and citrate cycle (TCA cycle) and other 8 metabolic pathways. These results suggest that resveratrol could affect several bacterial central metabolic pathways, such as TCA cycle in *A. hydrophila*.

### Expression trend analysis

**Figure 5:**
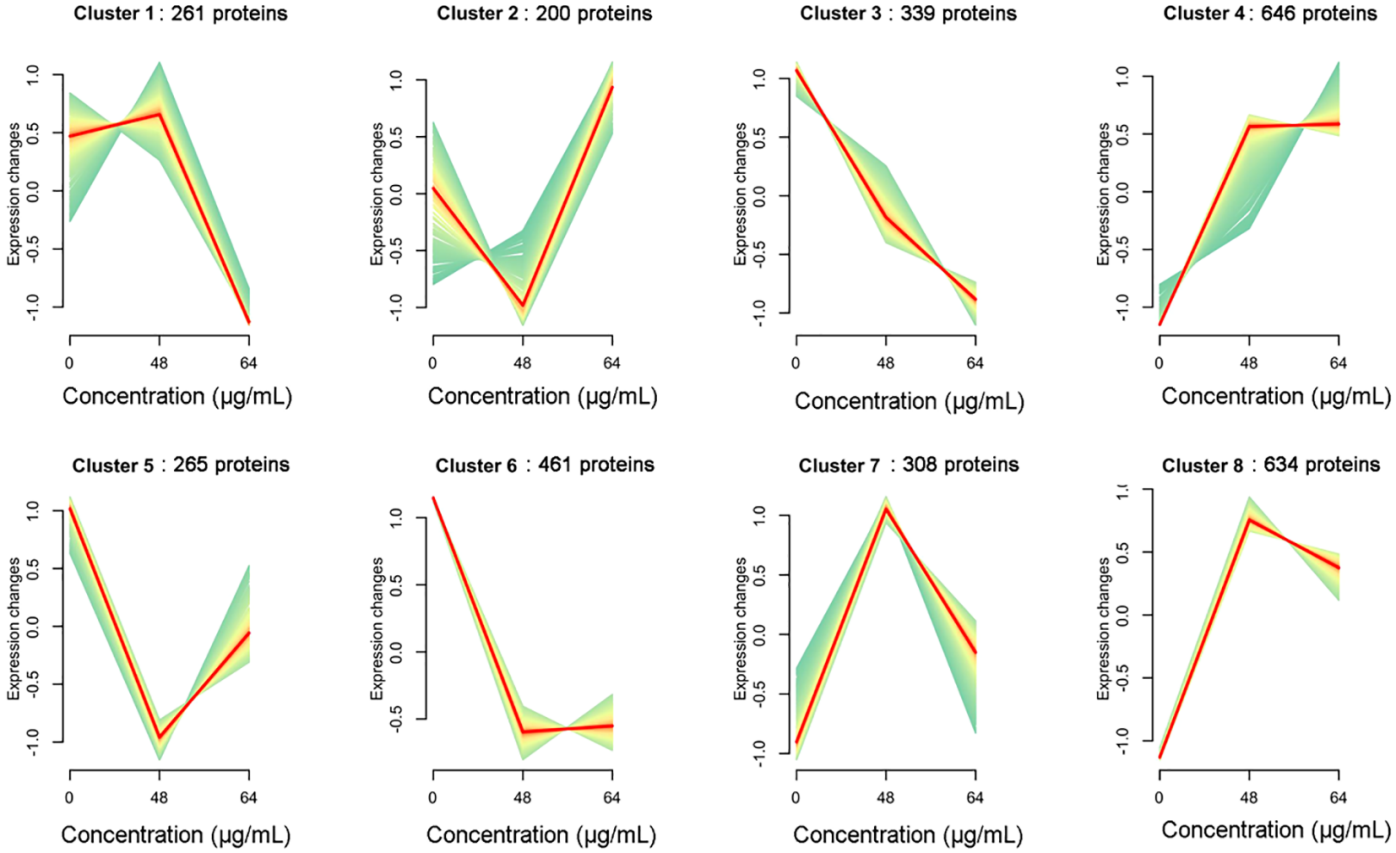
Dynamic trend analysis of DEPs under 48, 64 μg/mL resveratrol treatment. In a cluster, the light green line shows the trend of expression of each protein, and the red line shows the trend of one expression pattern.

We further analyzed the association between resveratrol concentrations and protein expression pattern in *A. hydrophila* in this study. According to the abundance of all proteins treated with different resveratrol concentrations, the gene clustering trend of these proteins was analyzed. As shown in **Figure 5**, a total of 8 clusters were obtained, which represented 8 expression patterns of proteins. With the increasing of Res concentrations, the expression of proteins in cluster 1 (261 proteins) was slowly up-regulated and then rapidly down-regulated, while the expression was immediate up-regulated after down-regulated in cluster 2 (200 proteins), and cluster 5 (265), but the expression changes differ each other. Expression changes of cluster 4 (646), cluster 7 (308) and cluster 8 (634) were increasing to 48 μg/mL Res, and then decreasing in 64 μg/mL Res. Compared to control that without Res, the expression of genes was down-regulated significantly with 48 μg/mL Res, and that was remained unchanged with 64 μg/mL Res. Unlike other expression patterns, cluster 3 was the only down-regulated pattern, involving 339 proteins, suggesting that the cluster proteins may play an important role in regulating gene expression.

### Validation of proteomic data at the mRNA level

**Figure 6:**
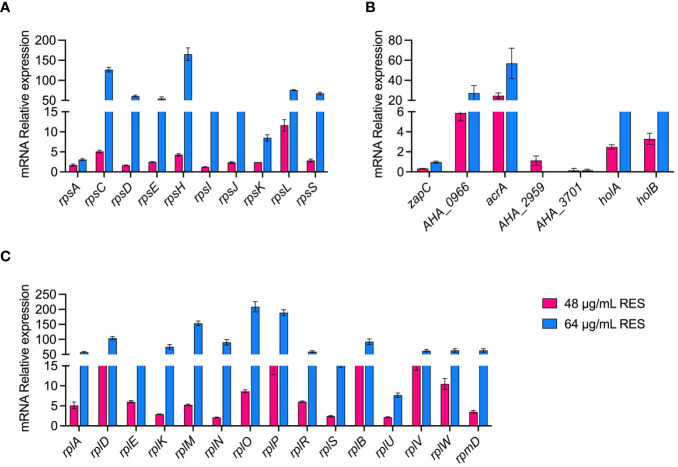
qPCR validation of *A. hydrophila* without or with 48, 64 μg/mL resveratrol treatment. **(A, B)** 30S ribosomal proteins; **(C, D)** 50S ribosomal proteins; **(E, F)** Other genes.

We verified the reliability of the proteomic data by measuring the expression of some genes at the mRNA level under 48 or 64 μg/mL resveratrol treatments by qPCR. As shown in **Figure 6**, a total of 25 ribosomal subunits (30S/50S) and 7 DEPs (*zapC, AHA_0966, araA, AHA_2959, AHA_3701, holA* and *holB*) that are increased abundance, and 3 decreased abundance (*zapC, AHA_2959, AHA_3701*) DEPs in proteomics results were selected for verification in mRNA level by qPCR. The results showed that the experimental data of qPCR were basically consistent with the corresponding proteomic data, which further demonstrated the reliability and repeatability of the omics data.

### The effect of resveratrol on bacterial morphology

**Figure 7:**
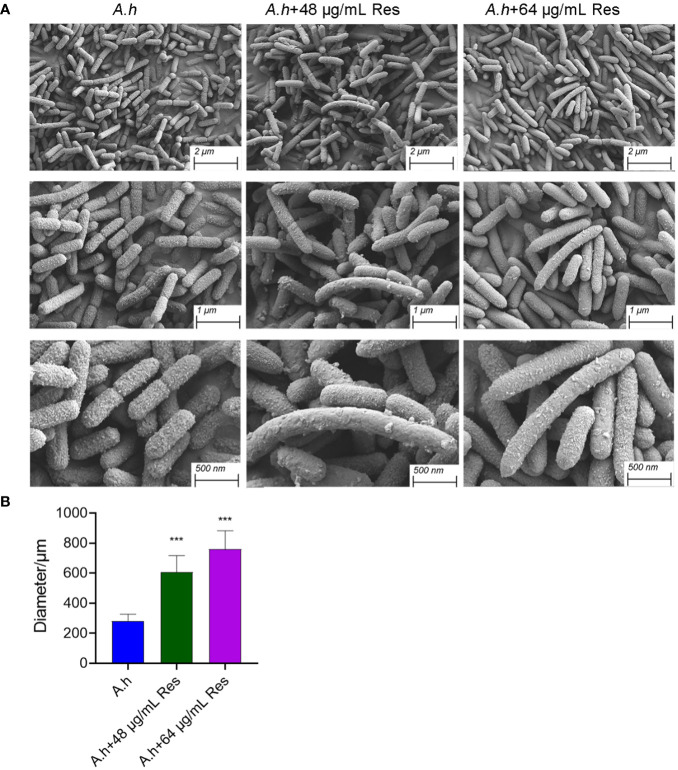
SEM results of *A*. *hydrophila* without or with 48, 64 μg/mL resveratrol treatment. **(A)** SEM photography of *A*. *hydrophila* cell without or with 48, 64 μg/mL resveratrol treatment; **(B)** The histogram of cell size. *** represents P<0.05.

In his study, SEM was performed to investigate the changes of morphology in *A. hydrophila* with and without the treatment of Res. As shown in **Figure 7**, Res treatment resulted in remarkable changes in a dose-dependent manner. Compared with the control group, the cell shape has barely changed, but cell size increased with the increasing Res concentrations. Untreated *A. hydrophila* single cell exhibited regular size that is about 250 μm in length, while approached about 600 and 750 μm in length under 48 or 64 μg/mL Res, respectively. The morphology change of *A. hydrophila* cells might be due to the effect of expression of cell division related genes were stressed by Res.

## Discussion

Owing to the escalating issue of antibiotic resistance due to misuse and overuse, the urgent quest for alternative antimicrobials is imperative. Natural plant extracts, known for their ability to disrupt pathogenic bacterial membranes and containing various beneficial compounds, emerge as potential alternatives (Santos-Zea et al., 2012; Lee et al., 2016; Peng et al., 2019). Resveratrol, an antimicrobial agent of plant origin, has garnered attention for its efficacy, low toxicity, and reduced drug resistance compared to conventional antibiotics (Wang et al., 2023b). It has been shown many beneficial effects in humans and animals, and widely used in the treatment of cancer and cardiovascular disease (Nøhr-Meldgaard et al., 2018). Moreover, resveratrol, including its derivatives, possess a potent antimicrobial activity against both Gram-negative and Gram-positive bacteria (Cebrián et al., 2023). Recently, numerous studies have shown that resveratrol has antibacterial effect on at least 20 kinds of bacteria, such as *E. coli* (Paulo et al., 2010), *Klebsiella pneumoniae* (Jung et al., 2009), and *Pseudomonas aeruginosa* (Nøhr-Meldgaard et al., 2018). However, limited research has explored its antibacterial impact on *A. hydrophila*.

This study treated A. hydrophila with Res at concentrations of 48 and 64 μg/mL, employing proteomics analysis to unveil alterations in protein expression. A total of 1049 proteins were identified under treatment with 48 μg/mL Res, including 607 up-regulated and 491 down-regulated proteins. Similarly, 562 up-regulated and 474 down-regulated proteins were found under the treatment with 64 μg/mL Res. Subsequent bioinformatics analysis identified significant changes in metabolic pathways, particularly those associated with cell wall biogenesis/degradation and peptidoglycan synthesis, suggesting a potential link to biofilm formation inhibition (Zohbi et al., 2014; Marti et al., 2017). For instance, Julianna et al., demonstrated that the cell wall biogenesis/degradation pathway can impede biofilm formation leading to bacteriostatic effects (Moraes et al., 2014). Our current study displayed the Res could inhibit *A. hydrophila* biofilm formation under resveratrol treatment, emphasizing the role of the cell wall biogenesis/degradation pathway in hindering biofilm formation.

Additionally, resveratrol has been reported to affect not only key metabolic pathways, but also the expression of key genes to produce antibacterial effects (Vestergaard and Ingmer, 2019). In our study, we observed a significant down-regulation in the expression of *zapC* gene related to cell division under resveratrol treatment suggesting that this antibacterial agent may exert its antimicrobial activity through inhibiting ZapC protein expression. The cell division protein ZapC has been reported to exert an ATP-dependent destabilizing effect on FtsZ polymerization *in vitro*, which is a crucial process for membrane formation during cell division mediated by the GTPase FtsZ (Hale et al., 2011; Marteyn et al., 2014; Du and Lutkenhaus, 2019). mRNA expression analysis and electron microscopy further validated the down-regulation of *zapC* and revealed resveratrol’s promotion of cell division, providing comprehensive insights into its antibacterial mechanisms. This multifaceted approach enhances our understanding of resveratrol’s potential as an alternative antimicrobial against *A. hydrophila*.

## Data availability statement

The mass spectrometry proteomics raw datasets generated for this study have been deposited to the ProteomeXchange Consortium via the iProX partner repository with the dataset identifier PXD048957 (https://www.iprox .cn/page/project.html?id=IPX0008094000).

## Author contributions

YF: Conceptualization, Data curation, Funding acquisition, Methodology, Writing – original draft. LZ: Conceptualization, Methodology, Software, Validation, Visualization, Writing – original draft. YL: Methodology, Writing – original draft. XZ: Conceptualization, Supervision, Writing – original draft. HC: Validation, Writing – original draft. YZ: Methodology, Writing – original draft. WJ: Formal analysis, Writing – original draft. XW: Data curation, Writing – original draft. XL: Conceptualization, Funding acquisition, Project administration, Supervision, Writing – review & editing.
