# Supplementary material for: Unveiling the antibacterial mechanism of resveratrol against Aeromonas hydrophila through proteomics analysis
Source: Front Cell Infect Microbiol. 2024 Mar 6;14:1378094. doi: 10.3389/fcimb.2024.1378094 (PMC10951904; doi:10.3389/fcimb.2024.1378094)
Supplement: Supplementary file 1 [file Table_1.docx]

**Supplementary Table S1. The primer pairs used in this study.**

| Gene | Primer | Base sequence (5’-3’) |
| --- | --- | --- |
| *rpsA* | qPCR*-rpsA*-F | GGTATCTTCATCGGTCTGGAT |
|  | qPCR-*rpsA*-R | CCCTTCACAATAGCACCTTTCT |
| *rpsC* | qPCR-*rpsC*-F | GCTGGAGCGTCGTGTAATGT |
|  | qPCR*-rpsC*-R | GGGCTTCAGAAGTTGCGTAG |
| *rpsD* | qPCR-*rpsD*-F | CCGTATGGGCTTCGGTGCTA |
|  | qPCR-*rpsD*-R | GACAGGTCGGAACGCTCTGG |
| *rpsE* | qPCR-*rpsE*-F | TTGACCGTGGTGGGTGATGG |
|  | qPCR-*rpsE*-R | GGTGGTGCAGAGTGCCGTTG |
| *rpsH* | qPCR-*rpsH*-F | AGGTTACATCACTGGCTACTCC |
|  | qPCR-*rpsH*-R | CCCATAACTTTCGGCAGATC |
| *rpsI* | qPCR-*rpsI*-F | ATTTATCAAAGCGGGTAGCG |
|  | qPCR-*rpsI*-R | CCAGTTTCTCGGTCATCTCAA |
| *rpsJ* | qPCR-*rpsJ*-F | TCGGGCGGTGTTGATTTATG |
|  | qPCR-*rpsJ*-R | GAGTCGGCAGCGGAATAGGA |
| *rpsK* | qPCR-*rpsK*-F | CTGTCATGGGCTACCTCTGG |
|  | qPCR-*rpsK*-R | CTTCACGCCGTATTCTTTGG |
| *rpsL* | qPCR-*rpsL*-F | TTCGCAAGCCACGCATCAAG |
|  | qPCR-*rpsL*-R | GGCACAGTCCAACGCACCAC |
| *rpsS* | qPCR-*rpsS*-F | TTTGACCATCGCTGTCCATA |
|  | qPCR-*rpsS*-R | GAACGTGCTGACGACCATTA |
| *rplA* | qPCR-*rplA*-F | TCTGATGCCGAACCCGAAAG |
|  | qPCR-*rplA*-R | TGGTGGTGGAGATGCTGACT |
| *rplD* | qPCR-*rplD*-F | GCTAAGCCGCAAGATCACAG |
|  | qPCR-*rplD*-R | TGCCGAACTTCTCAACAACG |
| *rplE* | qPCR-*rplE*-F | TGTTGCGGGCTTCAAGATTC |
|  | qPCR-*rplE*-R | CGGATACGCGGTACGGAGAT |
| *rplK* | qPCR-*rplK*-F | GTTTACAGCGACCGTTCCTT |
|  | qPCR-*rplK*-R | GAGCCACAGTCACCTTACCA |
| *rplM* | qPCR-*rplM*-F | CCTCCCGTTGGTCCTGCTCT |
|  | qPCR-*rplM*-R | TGGTCTTGGCGATTTCTTGC |
| *rplN* | qPCR-*rplN*-F | CGGTAAAGTGAAGAAAGGTGA |
|  | qPCR-*rplN*-R | CATTGTTGTCGAAACGGATG |
| *rplO* | qPCR-*rplO*-F | CGGTTTCTTCTCCCGCAAAT |
|  | qPCR-*rplO*-R | ACCACGAACGGTCACAGCAC |
| *rplP* | qPCR-*rplP*-F | GTACTAAATTCCGCAAGACCC |
|  | qPCR-*rplP*-R | CCTTACCTTTACCCATACGAAC |
| *rplR* | qPCR-*rplR*-F | GCTCGTCTCCGTCGTGCTAC |
|  | qPCR-*rplR*-R | GCGATGCGACCGTGATACTT |
| *rplS* | qPCR-*rplS*-F | CTTTCACCGTTCGCAAGATC |
|  | qPCR-*rplS*-R | ATCACCACGACGCTTCAGTT |
| *rplU* | qPCR-*rplU*-F | CGTTGAGACTGGCGCTACCA |
|  | qPCR-*rplU*-R | CGACGACGGAACTTGACGAT |
| *rplV* | qPCR-*rplV*-F | ACACCGTTATGCCCGTACTTC |
|  | qPCR-*rplV*-R | TTCGTTGTGCTCAGCGTTTG |
| *rplW* | qPCR-*rplW*-F | ATGATCCGTGAAGAGCGTCTG |
|  | qPCR-*rplW*-R | CTTTGACTTCCGCCTTGGTG |
| *rpmD* | qPCR-*rpmD*-F | CAAACTCGCAGCTCTATCGG |
|  | qPCR-*rpmD*-R | AGCCCTCCACCTTAACCATG |
| *zapC* | qPCR-*zapC*-F | CGAACTGGAGTGGAGCGTAC |
|  | qPCR-*zapC*-R | GTGTCTTGCTGGTGGTCAAT |
| *zapE* | qPCR-*zapE*-F | AGGCAAGACCTGGCTGATGG |
|  | qPCR-*zapE*-R | GCGTCGGTGATGTCGGAGAC |
| *AHA_0966* | qPCR-*AHA_0966*-F | CCCAGGCAAAGCAGAACAAGC |
|  | qPCR-*AHA_0966*-R | TCAGGGATTCGGTGGAGACG |
| *acrA* | qPCR-*acrA*-F | GCCGACAGCCTCACTTCCAT |
|  | qPCR-*acrA*-R | CTTTCTGCTCGTCCTGGGTCA |
| *AHA_2959* | qPCR- *AHA_2959*-F | GCCGAACTCAACCTCAGCTACA |
|  | qPCR-*AHA_2959*-R | TTGGCCTCAATCAATCCCTTT |
| *AHA_3701* | qPCR-*AHA_3701*-F | CACTGCCGATGAGTTGATGC |
|  | qPCR-*AHA_3701*-R | TTCTTGTTGCTCCGACTTGC |
| *holB* | qPCR- *holB* -F | GCAAGGGCTGGACTGGAATC |
|  | qPCR-*holA*-R | TCATCTGGGTCTGGTTGAGGC |
| *holB* | qPCR-*holB*-F | GCCTTGTGGTCAATGTCACTCC |
|  | qPCR-*holB*-R | GCAGGCTCTTCCAGGGTTTT |
